# Mental Health in Primary Care: Increasing Public and Patient Involvement in Health Research. Selected Abstracts from the EGPRN meeting in Dublin, Ireland, 19–22 October 2017

**DOI:** 10.1080/13814788.2018.1492538

**Published:** 2018-09-26

## Introduction to the theme of the conference

The central role of primary care within the field of mental health is a global phenomenon with policymakers actively encouraging primary care to take a lead role in developing and delivering mental health services.

Involving patients actively in research represents a significant culture change. Public and patient involvement (PPI) describes a whole variety of ways that researchers engage with people for whom their research holds relevance. The challenge is to mainstream PPI in all aspects of research, health policy and structures going forward, which will require significant political leadership and a new overall strategy.

