# Supplementary material for: Mental Health in Primary Care: Increasing Public and Patient Involvement in Health Research. Selected Abstracts from the EGPRN meeting in Dublin, Ireland, 19–22 October 2017
Source: Eur J Gen Pract. 2018 Sep 26;24(1):211–22. doi: 10.1080/13814788.2018.1492538 (PMC6161616; doi:10.1080/13814788.2018.1492538)
Supplement: Related texts

# Abstract: Increasing public and patient involvement in research—methods, motivations and the particular issues for mental health projects

**Authors:** Amanda Howe

**Affiliations:** Faculty of Medicine and Health Sciences Primary Care, University of East Anglia, UK

## Abstract

Clinical research relies on patients being willing to participate in research projects, and making this possible for patients with mental health problems can be a particular challenge. In the modern era, many countries have seen a movement to give a stronger voice to patients both in choices around their care and in how research is conducted. How to achieve effective patient and public involvement (PPI) and to make the patients real partners in this effort is itself a subject of research evaluation. This opinion piece—based on a keynote lecture given at the European General Practice Research Network 2017 autumn meeting in Dublin—describes both the reasons for expanding PPI, how it can usefully be achieved, and how this may relate to the particular context of mental health. There can be moral, methodological or policy reasons for PPI. The three commonest models of good practice in PPI are the ‘one off,’ the ‘fully embedded’ and the ‘outreach’ models. In research into common mental health problems in family practice, ‘outreach’ approaches that minimize commitment over time may work best. ‘Expert patients’ from mental health charities can sometimes play this role. PPI may be challenging and involve extra effort, but the gains for all may be considerable. WONCA Europe networks including EGPRN can extend this message and findings.

An article based on this keynote lecture was published in the *European Journal of General Practice* as: Howe A. Principles of patient and public involvement in primary care research, applied to mental health research. A keynote paper from the EGPRN Autumn Conference 2017 in Dublin. Eur J Gen Pract. 2018;24:167–170. https://doi.org/10.1080/13814788.2018.1470620

# Abstract: New developments in Ireland with regard to the clinical trial network for primary care

**Authors:** Andrew Murphy

**Affiliations:** Department of General Practice, National University of Ireland, Galway

## Abstract

In 2015 the Health Research Board (HRB)—the main funder of Irish health research—awarded €2.5 million each to four trial networks. One of these was in primary care. In this presentation, the international background, general role and development of research networks in primary care will be reviewed. The context, vision and progress to date of the HRB Clinical Trial Network Primary Care Ireland will be outlined. Consideration of the challenges and opportunities facing international primary care research networks will occur. An invitation to international colleagues to collaborate with the Irish network will be warmly issued.[Fig F0001]

**Figure F0001:**

# Abstract: Hepatitis C management among patients receiving opioid substitution treatment in general practice in Ireland

**Authors:** Ross Murtagh, Davina Swan, Eileen O’Connor, Geoff McCombe, Carol Murphy, John S. Lambert, Gordana Avramovic, Walter Cullen

**Affiliations:** University College Dublin, Mater Misericordiae University Hospital, Dublin, Ireland

## Abstract

**Background:** Hepatitis C (HCV) infection is associated with considerable morbidity and public health burden. Injecting drug use is the main route of transmission in most countries. In Ireland and other European countries, general practice is increasingly providing continuing care, including opioid substitution treatment (OST), for people who inject drugs (PWID). Thus, general practice is well placed to care for PWID at risk of and infected with HCV.

**Research question:** We aim to describe the current management of HCV among patients on OST in general practice in Ireland.

**Methods:** OST-prescribing GP practices in North Dublin were recruited from the professional networks/databases of the research team. Patients were eligible to participate if ≥18 years of age, on OST, and attend the practice for any reason during the recruitment period. A standardized non-probability sampling framework was used to identify 10 consecutive patients from each practice to participate. The clinical records of participants were reviewed and data extracted from demographics and HCV-related care processes. Descriptive statistical analysis was performed using SPSS V24.

**Results:** Fourteen practices and 135 patients were recruited. Data was extracted from the charts of 133 patients; 71.4% male, median age 42 years. There was evidence that 92.5%, 72.2%, and 66.9% had been screened for HCV, HIV, and hepatitis B (HBV), respectively. Among those who had been tested, the prevalence of HCV, HIV, and HBV infection was 77.2%, 8.3%, and 7.9% respectively. Just 14 (14.7%) patients previously diagnosed with HCV had ever initiated HCV treatment. In the past year, 24.8% of patients had been asked about their alcohol use by their GP, 5.3% had received a brief intervention, and 2.3% had received an alcohol-related referral.

**Conclusion:** Most OST patients had been screened for HCV. However, a minority had initiated HCV treatment. New strategies are needed to facilitate greater continuity of care among this population.

# Abstract: Patient views on urinary tract infection and outcome measures in studies and guidelines on urinary tract infection

**Authors:** Eva Hummers-Pradier, Michaela Aschenbrenner, Ildikó Gágyor, Jutta Bleidorn, Susanne Heim, Guido Schmiemann

**Affiliations:** University Medical Centre Göttingen, Institute of General Practice, Göttingen, Germany

## Abstract

**Background:** Authors of guidelines or proposals for clinical trials are increasingly requested to prove that their outcome measures or recommendations meet patients’ concerns and priorities. Consulting patient representatives is often mandatory when preparing a study proposal for public funding programmes.

**Research question:** To assess the patient relevance of outcomes of a planned—now ongoing—urinary tract infection (UTI) trial well as in the national guideline, and to investigate patients’ concerns and priorities related to UTI.

**Methods:** We (1) systematically reviewed outcome measures used in UTI trials, (2) systematically searched for papers on patients’ views and concerns on UTI and its treatment and (3) performed four focus groups with UTI patients to discuss their views. Focus groups were videotaped, transcribed, and analysed using qualitative content analysis.

**Results:** Most UTI trials feature either urine cultures and/or typical symptoms as primary outcome measures. Symptoms are often assessed by doctors or nurses, rather than patients, and scored nominally or they are assessed using simple symptom scores that are mostly not validated. Trials considering in detail how much patients are actually impaired by their condition are rare. Few qualitative studies look more in-depth into patient perceptions and priorities show a more differentiated picture. Focus group participants perceived UTI as complex and named a multitude of relevant topics with regard to expectations, feelings, restrictions, treatment, counselling and follow-up.

**Conclusion:** Patients can be severely impaired and very concerned with many aspects of UTI. Many UTI trials do not consider patient relevant outcomes at all, or only with a relatively coarse approach. Many of patients’ priorities are reflected neither in guidelines nor in studies.

# Abstract: Why is the physical health of patients with an enduring mental illness neglected? Comparing Irish GPs’ monitoring of physical health parameters among patients with an enduring mental illness and the general population

**Authors:** Claire Collins, James Larkin, Ivana Pericin, Brian Osborne, Philip Dodd

**Affiliations:** Irish College of General Practitioners, Dublin, Ireland

## Abstract

**Background:** People with enduring mental illness (EMI)—schizophrenia, bipolar disorder and/or (recurrent) depressive disorder—have a mortality rate two-to-three times higher than the general population, which is caused by physical disease. This is thought to be primarily caused by diagnostic overshadowing, whereby healthcare staff attributes symptoms of physical illness to their EMI. Diagnostic overshadowing thus leads healthcare practitioners to focus less on the physical illnesses of people with EMI and more on their mental illness. This study will compare Irish GPs’ recording of physical health parameters among EMI patients and the general population.

**Research question:** Is there a difference in Irish GPs’ monitoring of physical health parameters among patients with an EMI compared to the general population?

**Methods:** This is a retrospective case-control study. Data will be extracted from the practice management software systems of 87 Irish GPs on physical health parameter recording of EMI patients and the general population over a 12-month period. These parameters will include alcohol consumption, physical activity, BMI, smoking status and brief interventions. Practices will come from a mixture of urban and rural areas across Ireland. Difference in recording of EMI patients will be compared to the general population using ANOVA.

**Results:** No results yet. A possible outcome is that GPs record physical health variables amongst patients with an EMI less than with the general population.

**Conclusion:** Lack of recording of physical health variables may contribute to the large discrepancy in life expectancy between EMI patients and the general population. Introducing a systematic means of recording physical health parameters to GPs’ practice management systems could improve EMI patients’ care and thus reduce the discrepancy in life expectancy between EMI patients and the general population.

# Abstract: ‘Working away in that Grey Area…’ A qualitative exploration of the challenges General Practitioners experience when managing behavioural and psychological symptoms of dementia

**Authors:** Aisling Jennings, Tony Foley, Sheena McHugh, John Browne, Colin Bradley

**Affiliations:** University College Cork, Cork, Ireland

## Abstract

**Background:** General practitioners (GPs) have identified the management of behavioural and psychological symptoms of dementia (BPSD) as a particularly challenging aspect of dementia care. However, no research to date has explored the challenges GPs experience managing BPSD or how these challenges influence the care GPs offer to their patients with dementia.

**Research question:** To establish the challenges GPs experience managing BPSD; to explore how these challenges influence GPs’ management decisions; and to identify strategies for overcoming these challenges.

**Methods:** This was a qualitative descriptive interpretive study of GPs experiences of managing BPSD. Semi-structured interviews were conducted with 16 GPs in the Republic of Ireland. Nursing homes were contacted and the GP who attended the nursing home was identified. From this population, a sample was purposively selected to include GPs with differing practice locations (urban/rural), years in practice and dementia workload. GPs with no nursing home commitment but who cared for people with dementia in the community were also purposively recruited to provide maximum diversity of views. Interviews were analysed thematically by two independent reviewers.

**Results:** Three main challenges of managing BPSD were identified; lack of clinical guidance, stretched resources and difficulties in managing expectations. The lack of appropriate clinical guidance, which referred to both the lack of guidelines and difficulty accessing clinical advice, meant that the GPs relied on their own experiences of the drugs when making prescribing decisions. In the absence of adequate resources, GPs felt reliant upon sedative medications. Finally, GPs believed their advocacy role was further compromised by the difficulties they experienced managing expectations of family caregivers and nursing home staff.

**Conclusion:** This study helps to explain the apparent discrepancy between best practice recommendations in BPSD and real-life practice. It will be used to inform the design of an intervention to support the management of BPSD in general practice.

# Abstract: Detection of cognitive decline in General Practitioner (GP) office

**Authors:** Miroslav Hanževački

**Affiliations:** Health Care Center, Zagreb West, Zagreb, Croatia

## Abstract

**Background:** Although cognitive decline is a common finding among the elderly and is considered a risk factor for developing dementia, it is rarely diagnosed by general practitioners (GPs).

**Objectives:** To evaluate cognitive function with the Mini-Mental State Examination (MMSE) and the Montreal Cognitive Assessment (MoCA) in asymptomatic subjects in daily GP practice and compare subjects who confirmed having cognitive problems with subjects who did not claim any cognitive problem.

**Research question:** Can we detect and evaluate mild cognitive decline already in general practice?

**Methods:** 388 consecutive patients >65 years of age in daily GP practice were interviewed and tested with MMSE and MoCA tests.

**Results:** None of the study subjects spontaneously complained of cognitive or memory problems, 155 subjects (39.94%) confirmed having cognitive problems and 233 (60.05%) did not, even when asked. The prevalence of mild cognitive impairment (MCI) was 18.30% (95%CI: 14.36–22.04) and the prevalence of cognitive impairment/no dementia (CIND) was 17.27% (95%CI: 13.50–21.04). Delayed memory recall as a separate cognitive domain in MoCA was significantly worse in subjects with MCI (*P* = 0.00958) and those with CIND (*P* = 0.0208).

**Conclusion:** Mild cognitive decline can be detected already in GP office by using simple screening tests MMSE and MoCA. Most patients with cognitive decline do not claim any problem and do not seek active help for this reason.

# Abstract: Higher all-cause mortality and accelerated cognitive decline in the oldest-old with low blood pressure and antihypertensive treatment—results from a population-based Dutch cohort study

**Authors:** Sven Streit, Rosalinde Poortvliet, Jacobijn Gussekloo

**Affiliations:** a Institute of Primary Health Care (BIHAM), University of Bern, Bern, Switzerland;; b Department of Public Health and Primary Care, Leiden University Medical Center, Leiden, the Netherlands

## Abstract

**Background:** In the oldest-old (> 80 years), trials showed efficacy of lowering systolic blood pressure (SBP) including mostly healthier individuals but observational studies raised concerns about associations of low SBP with increased mortality and negative effects on mental health (e.g. cognition) especially in older and frail patients.

**Research question:** To test if the association of low SBP with mortality and cognitive function is modified by antihypertensive treatment and if associations differ in frail patients.

**Methods:** This prospective cohort study (Leiden 85-plus Study, 1997–2004) included a population-based sample of 570 individuals aged 85 years with a five-year follow-up for cognitive function and all-cause mortality. Exposure was SBP at baseline (per 10 mmHg lower) for patients with/without antihypertensive treatment. We modelled Cox proportional hazards for all-cause mortality and mixed-effect linear regressions for change in Mini-Mental State Examination (MMSE) adjusted for sex and history of cardiovascular disease (CVD). In subgroup analyses, we stratified for frail/non-frail patients according to handgrip strength and the Groningen Activity Restriction Score (GARS).

**Results:** Of all participants, 249 (43.7%) were treated with antihypertensives at baseline. CVD was more prevalent in those with treatment (*P* <0.001). In patients with treatment, all-cause mortality was higher (HRAdj 1.30; 95%CI: 1.15–1.47; *P* <0.001) per 10 mmHg lower SBP. Similarly, per 10 mmHg lower SBP, patients showed an accelerated cognitive decline (–0.35; 95%CI: –0.60 to –0.11; *P* = 0.004). In patients without treatment, there were no such associations. Patients frail in handgrip showed the same accelerated cognitive decline (–0.37; 95%CI: –0.70 to –0.05; *P* = 0.023) but non-frail patients did not (*P* = 0.15). Stratification based on GARS did not influence the relationship.

**Conclusion:** The oldest-old with low SBP and antihypertensive treatment are at risk for higher all-cause mortality and accelerated cognitive decline especially frail patients. Although observational and limited by confounding by indication, strengths of associations, temporality and biological gradient support causal interpretations.

# Abstract: What is the internal validation and dimensionality in the translation of HSCL-25 in French, in the diagnosis of depression in primary care?

**Authors:** Patrice Nabbe

**Affiliations:** Faculté de Médecine et des Sciences de la Santé, EA SPURBO 7479/Département Universitaire de Médecine Générale, BREST CEDEX 3, France;

## Abstract

**Background:** Diagnosis of depression is difficult and diagnostic tools are rarely used by GPs. The Hopkins Checklist Symptom in 25 items (HSCL-25) was retained to fill this gap. It is a clinical tool, validated against a psychiatric examination according to Diagnostic and Statistical Manual of Mental Disorders (DSM) major depression criteria. Following a RAND/UCLA, the HSCL-25 has been selected as the most efficient, reliable and ergonomic tool combined. HSCL-25 has been translated into French using a forward/backward translation according to Delphi procedure.

**Research question:** The last phase consisted of comparing HSCL-25 scale against Patient State Examination-9 version (PSE-9); it has confirmed the internal validation and dimensionality of the French HSCL-25 version in primary care.

**Methods:** A quantitative study comparing HSCL-25 and PSE-9, in several medical centres, for adult patients considered depressed or not by the two tools. Adult patients who completed ethical consent were selected. Patients lived in Finistère (France) in 2015. A patient is considered ‘depressive’ if her/his mean HSCL-25 score is greater than or equal to 1.75. In the HSCL– group, one in 16 patients have performed PSE-9 while in the HSCL + group, the ratio is 1:2.

**Results:** Patients involved met the inclusion criteria. After removing duplicates and wrong inclusions, 1126 of 1134 patients were included. The factor analysis showed that the HSCL-25 tool is one-dimensional—which combined anxiety and depression dimensions—with a Cronbach alpha of 0.93.

**Conclusion:** The HSCL-25 scale has a high eigenvalue. A one-dimensional tool combined items according to anxiety and depression. This reliable tool will assist primary care in daily practice.

# Abstract: What factors are associated with antidepressant and benzodiazepine prescribing to people with type two diabetes mellitus?

**Authors:** Jane O’Doherty, Jennifer Gettings, Raymond O’Connor, Ailish Hannigan, Walter Cullen, Louise Hickey, Andrew O’Regan

**Affiliations:** University of Limerick, Limerick, Ireland

## Abstract

**Background:** General practitioners (GPs) are responsible for diagnosing and treating people with type 2 diabetes mellitus (T2DM) and co-morbid depression and anxiety. The prevalence of depression in T2DM is estimated to be 10–15% but there is no data on the proportion of people with T2DM that are prescribed antidepressant or benzodiazepine medications. This study aimed to investigate patients with T2DM who were prescribed antidepressant or benzodiazepine medications. A secondary objective was to establish the proportion that were referred to mental health services.

**Research question:** What percentage of people with T2DM are prescribed antidepressants or benzodiazepine medication?

**Methods:** Senior medical students on placement in general practices with the University of Limerick Graduate Entry Medical School and their GP supervisors used practice software functions to collect quantitative data from the clinical records of patients with T2DM in the practice.

**Results:** The sample included 2696 patients with T2DM who had visited their GP in the previous year. The percentage of people with T2DM with a current prescription for antidepressants or benzodiazepine medication varied from 6–37% across practices with a median rate across practices of 23%. Using logistic regression analysis, the following were found to be associated with having this type of prescription: female gender, eligibility for free medical care, urban location, smoking, and consulting with the GP more frequently. Only 6% (*n* = 152) of patients were referred or attended a mental health service.

**Conclusion:** This study shows that the rate of antidepressant and benzodiazepine prescribing in a T2DM population is high. Based on the findings, screening for depression in this population is advised and appropriate resources for GPs should be provided.

# Abstract: Too depressed to sleep: Long-term hypnotic treatment and mental illness. A 10-year retrospective cohort study of 250 000 patients

**Authors:** Yochai Schonmann, Or Goren, Ronen Bareket, Doron Comaneshter, Arnon Cohen, Shlomo Vinker

**Affiliations:** Clalit Health Services, Department of Family Medicine, Rabin Medical Center, Aviv, Israel

## Abstract

**Background:** Mental illness is thought to be associated with an increased risk of benzodiazepine abuse, but long-term data is limited, especially in the context of insomnia treatment.

**Research question:** Are community-dwelling patients with mental conditions at an increased risk of long-term hypnotic use? Does the choice of hypnotic influence future outcomes?

**Methods:** A historic cohort analysis of all incident hypnotic medication users aged 21 and over, who were members of Clalit Health Services (Israel’s largest health provider). Information on background mental conditions, socio-demographic characteristics and initial hypnotic used was retrieved from the unified electronic health record. Amount of benzodiazepine hypnotics and Z-drugs purchased was ascertained on the second, fifth and tenth years. Logistic regression was used to determine predictors of long-term usage patterns among all participants, and specifically among those with a mental condition.

**Results:** A total of 254 239 patients initiated treatment for insomnia between 2005 and 2015. Mean age was 63.6 (SD 16.4) and 58.6% were women. A pre-existing mental condition was present in 74 543 (29.3%) of the patients. On the tenth year, 9408 (22.4%) of living patients with a mental condition where chronic hypnotic users and 336 (0.8%) took two pills a day or more. Risks of chronic- and excessive-use were higher for participants with a mental condition (OR =1.17; 95%CI: 1.13–1.20; *P* <0.0001, and OR =1.53; 95%CI: 1.31–1.78; *P* <0.0001, respectively). Predictors of chronic- and excessive-use among mental patients included antidepressant treatment (OR =1.13; 95%CI: 1.05–1.21; *P* = 0.0007, and OR =1.68; 95%CI: 1.24–2.29; *P* = 0.0008, respectively). Bipolar disorder (OR =1.11; 95%CI: 1.0.89–1.39; *P* = 0.3578, and OR =2.21; 95%CI: 1.16–4.23; *P* = 0.0313, respectively), initial Z-drug treatment (OR =1.52; 95%CI: 1.45–1.60; *P* <0.0001, and OR =2.31; 95%CI: 1.84–2.90; *P* <0.0001, respectively), but not schizophrenia (*P* = 0.5828 and 0.7552, respectively).

**Conclusion:** Mental patients are at increased risk of chronic hypnotic use, although absolute risk of excessive use is small. Z-drugs do not seem to confer a beneficial effect over benzodiazepine hypnotics in reducing long-term use.

# Abstract: Clients’ views on primary healthcare after detoxification for alcohol use disorders

**Authors:** Lodewijk Pas, Trudy Bekkering, Evelien Lenaerts, Cathy Mathei, Bert Aertgeerts

**Affiliations:** Academisch Centrum Huisartsgeneeskunde, Public Health & Primary Care, Leuven, Belgium

## Abstract

**Background:** To develop an experimental care pathway for alcohol-dependent patients we systematically reviewed the literature for continuing care models, defined indicators for quality of care after detoxification through a Delphi study, and finally have run focus groups to define principles for proper quality aftercare. The systematic review and indicators have been described elsewhere. Eight focus groups (FGs) were run to define barriers and facilitators for success of aftercare, two of which were held with lay people, the others with professional care providers.

**Research question:** What, according to lay people, are the major barriers and facilitators for effective aftercare?

**Methods:** A mixed female and male opportunity sample was constituted for two FGs of patients in aftercare in one location but with experience from different treatment facilities, some with their spouses. A moderator favoured discussion between participants according to a pre-established guidebook assisted by an observer to register the group process. The FGs were audio-taped and transcribed verbatim, independently coded by two researchers and analysed with NVivo software based on consensus and using a constant comparative approach.

**Results:** Factors favouring success of continuing care indicated by patients and their spouses are: (1) increasing insight of patients in the longstanding need for support early in care; (2) openness in a continued experiences oriented peer group process facilitated by a professional care provider; (3) availability of continued professional individual support during the aftercare peer group process; (4) information and involvement of the GPs before and early during aftercare in mental health facilities; (5) specific attention and, if possible, involvement of spouse or surroundings; (6) specific attention to underlying problems leading to alcohol use.

**Conclusion:** According to alcohol-dependent patients more active involvement of GP and surroundings in collaborative care is required and attention to the long-lasting character of alcohol use disorders and underlying societal, relational and individual problems.

# Abstract: Description of patients suffering from eating disorders managed by French GPs

**Authors:** Jean Sébastien Cadwallader, Massimiliano Orri, Christine Hassler, Caroline Barry, Bruno Falissard, Caroline Huas

**Affiliations:** University Pierre et Marie Curie, General Practice, UMR CESP, Paris, France

## Abstract

**Background:** Anorexia nervosa (AN) showed the highest mortality rates of all psychiatric disorders. Bulimia nervosa (BN) was also associated with an increased mortality rate. Incidence and prevalence rates of eating disorders (EDs) diagnosed in primary care studies were much lower than in the general population. GPs were often viewed in the secondary care literature as the best stakeholders to screen and manage ED patients at an early stage.

**Research question:** Who are patients with ED attending French GPs? What is the temporality between EDs and depression?

**Methods:** French cohort study including 1310 patients with at least one consultation about EDs. The database was the GPO (general practice observatory), based on the follow-up of 700 000 patients followed by 250 GPs between 1994 and 2009. ICD-10 and DSM IV were used to classify ED. The patients included were described according to age, gender, frequency and type of consultations, general and specific to ED follow-up periods, type of ED. Data were built to study the temporality between depression and EDs.

**Results:** 82.67% patients were women (mean age of 35.19 years). Median follow-up length was 5.35 years. 41.6% had an AN diagnosis, 26.4% BN, 32% EDNOS. AN patients were older. 16.3% of ED patients had their diagnosis at the first consultation with GPs. 32.3% of ED patients had at least one consultation for depression. Patients with both diagnoses were more often and longer followed, 75% of ED patients were prescribed benzodiazepines. 18.4% of them were prescribed antidepressants, mostly escitalopram. The diagnosis of depression did not precede the diagnosis of ED in our sampling.

**Conclusion:** This was the first study in France in a GP setting about ED patients. The prevalence seemed very low and ED patients seemed different from hospitalized patients. They were mostly managed for depression and had a probable medication overuse.

# Abstract: Factors associated with attitudes towards professionalism in medical students at Faculty of Medicine Ljubljana, Slovenia

**Authors:** Anja Cerne, Davorina Petek, Polona Selic

**Affiliations:** Department of Family Medicine, Faculty of Medicine, University of Ljubljana, Ljubljana, Slovenia

## Abstract

**Background:** Medical students have some attitudes towards professionalism before matriculation but these have been shown to change during the study process.

**Research question:** To explore factors associated with attitudes towards professionalism in medical students and to identify if they were associated with basic personal traits or due to study process.

**Methods:** This study represents first-year data of a six-year longitudinal pragmatic research, taking place at the Faculty of Medicine in Ljubljana, Slovenia. A sample of 210 students of 418 enrolled (50.2%) in the fourth and sixth year in the academic year 2015/16 voluntarily participated. The big five-personality test (BFQ), the Personal Values List (LOV) and the professionalism assessment scale were administered together with the set of basic socio-demographic data. In multivariate modelling, the association between demographic characteristics, LOV, BFQ, and attitudes towards professionalism were analysed.

**Results:** There were 66.5% females, 38.1% of them were from the urban origin. Participants significantly differed from Slovenian norms in four of five personality traits, i.e. in energy (*P* <0.001), in friendliness (*P* = 0.024), emotional stability (v <0.001) and openness (*P* <0.001). The most important preference declared was self-esteem (z = 0.36), the least children (z = –0.49). In multivariate modelling, 34.5% of variance was explained (R2 = 0.345, F = 2.450, *P* <0.001)). Male gender (β = –0.16, *P* = 0.038) and rural origin (β = –0.23, *P* = 0.006) were negatively, receptivity (β ** **= 0.34, *P* <0.001) and the values food (β** **= 0.24, *P* = 0.006), home (β** **= 0.24, *P* = 0.009), work (β** **= 0.19, *P* = 0.021), freedom (β** **= 0.19, *P* = 0.021) and power (β** **= 0.24, *P* = 0.006), respectively were positively associated with attitudes towards professionalism.

**Conclusion:** At this phase, attitudes were found to be associated with both hereditable and social factors, the last ones possibly influenced by study process. Given that, decision makers at the Faculty of Medicine in Ljubljana were warned to reconsider the curriculum and capacity building in mentors, given that interactive learning could affect values and attitudes towards professionalism.

# Abstract: Developing accreditation for minor surgery in general practice: the Irish experience

**Authors:** Ailis Ni Riain, Claire Collins, Tony O’Sullivan

**Affiliations:** Irish College of General Practitioners, Dublin, Ireland

## Abstract

**Background:** Carrying out minor surgery procedures in the primary care setting is popular with patients, cost-effective and delivers outcomes at least as good as those performed in hospital. Our objective was to develop a valid, robust, workable accreditation system for general practitioners (GPs) undertaking community-based surgery in Ireland where no mandatory accreditation currently exists.

**Research question:** What are the essential elements of an accreditation system for minor surgery carried out by experienced GPs? Will introducing accreditation for minor surgery be acceptable to GPs?

**Methods:** Twenty-four GPs were recruited to the GP Network. Ten pilot standards were developed addressing GPs’ experience and training, clinical activity and practice supporting infrastructure and tested, using information and document review, prospective collection of clinical data and a practice inspection visit. A multi-modal evaluation was undertaken. Quantitative data collected was analysed using predictive analytic software (PASW). Krueger’s framework analysis approach was used to analyse the qualitative data. The ICGP Research Ethics Committee provided ethical approval.

**Results:** Nine GPs achieved all pilot standards at initial review, 14 subsequently completed corrective actions and one GP did not achieve accreditation. Feedback from the GPs and surveyors contributed to amendments and additions to the standards. An accreditation system with 14 standards and a supporting framework was developed.

**Conclusion:** This project demonstrates that it is possible to develop robust quality standards for community-based minor surgical procedures in the real-life setting over a short timeframe. Clinical leadership promotes mutual support, ensures relevance and promotes buy-in.

# Abstract: General Practitioners are not emigrating to further their training—Trend analysis of the career intentions of recent graduates of General Practice training in Ireland

**Authors:** Gerard Mansfield, Claire Collins, Ivana Pericin, James Larkin

**Affiliations:** Irish College of General Practitioners, Dublin, Ireland

## Abstract

**Background:** A lack of government support, a capitation-based GP contract from 1989 and an aging practice population has led to an increased demand on the GP workforce in Ireland. Consequently, a significant number of recent GP graduates are considering emigration or part-time employment. To investigate a worsening workforce crisis, the present study analysed the career plans of GP graduates in Ireland.

**Research question:** What are the present career intentions of the recent GP graduates in Ireland?

**Method:** A ‘Career Intentions’ survey was emailed in 2014, 2015 and 2017 to all GP training graduates in Ireland. The surveys aimed to investigate employment status at that time, any emigration plans and the factors identified as having influenced those critical decisions. The average response rate across all three survey years was 34.5%. The data was analysed using SPSS Statistics 23.

**Results:** The number of GP graduates who emigrated slightly increased over the years, accounting for 16.9% in 2014, to 19.2% in 2017. The results displayed that 50% of graduates who emigrated did so after the first year and 75% in the first two years. The most common reasons for emigration were ‘quality of life’ and ‘financial prospects.’ In 2014, 47.3% of graduates stated they intended to work part-time in five years. This rose to 51.3% in 2015, reaching 60% in 2017. The intention to work part-time was significantly related to gender (*P* < 0.001); female participants were more than twice as likely to plan to work part-time in five years compared to males.

**Conclusion:** The first and second years after graduation were the most critical for emigration. Interventions in this period may reduce the brain drain of Irish GPs. Part-time working is becoming more attractive and will have impacts on the availability of GPs. This should be considered in future workforce-planning modules.

# Abstract: What can be done to promote resilience in general practitioners?

**Authors:** Olivia Gonzalez, Aine Waters, James Keogh, Nora Kavanagh, Clare O’Neill, Genevieve McGuire, Maureen Kelly

**Affiliations:** Western Training Programme in General Practice, Galway, Ireland

## Abstract

**Background:** Medicine is a challenging career. There are reports of high rates of exhaustion, burn out and maladaptive coping strategies amongst doctors in general, and general practitioners (GPs) in particular. Accordingly, the issue of GP resilience is extremely topical. This research aimed to explore the ideas and opinions of GPs and doctors in GP training about resilience promotion.

**Research question:** What can be done to promote resilience in GPs?

**Methods:** This was a qualitative study involving face-to-face, semi-structured, recorded interviews with GPs and doctors in GP training. Data analysis was done by using a three-step process of open, axial and selective coding supported by detailed memos.

**Results:** Three themes emerged from data analysis namely: (a) within the person, (b) within the profession and society, and (c) within the healthcare system. Subthemes within the person were ‘what resilience means to me,’ ‘me as a doctor,’ and ‘me as a human.’ Within the profession and society there were four subthemes ‘traditional view of GP,’ ‘societal expectations of the GP,’ ‘formal supports,’ and ‘education and training.’ The healthcare system theme incorporated three subthemes ‘finances,’ ‘manpower’ and ‘relationship with the healthcare system.’

**Conclusion:** Identifying means to promote resilience in GPs involves an in-depth understanding of the threats and challenges to resilience from within the person, the profession and society and the healthcare system. Specific measures to promote resilience can be developed to counteract each of these identified challenges. It is anticipated that this research will help inform and shape future strategies and educational interventions aimed at promoting resilience in GPs.

# Abstract: The challenge of change and maintain healthy behaviours. Effectiveness and cost-effectiveness of coaching to empower people with knee osteoarthritis and improve their quality of life.

**Authors:** Anna Berenguera, Enriqueta Pujol-Ribera, Victòria Carmona-Terés, Mariona Pons-Vigués, Joana Moix-Queraltó

**Affiliations:** IDIAP JORDI GOL, Sabadell, Spain

## Abstract

**Background:** First-line treatment for knee osteoarthritis (KO) should be non-pharmacological (weight loss, physical activity and self-management of pain). Health coaching facilitates the achievement of health improvement and self- management for chronic conditions.

**Research question:** To analyse the effectiveness of a health coaching intervention on quality of life, pain, overweight and physical activity in patients with KO from 18 primary care centres of Barcelona.

**Methods:** Methodology from the Medical Research Council on developing complex interventions. Phase 1: intervention modelling and operationalization through a qualitative study using theoretical sampling with patients with KO and with primary care professionals. Phase 2: a community-based randomized clinical trial with 360 patients with KO with an intervention group (IG) and control group (CG) (180 per group). IG receives usual care plus 20-h health coaching and follow-up sessions. CG receives routine care. The primary outcome is quality of life (WOMAC). Data analyses: comparison of proportions between study groups.

**Results:** Phase 1: Results indicated the barriers and facilitators of the health behaviours and transformed into recommendations for the intervention design. Phase 2: preliminary results on effectiveness after the intervention are present comparing IG vs CG, 415 participants were included: 234 IG and 181 CG. Intervention helps improve quality of life (57% IG reduce five points in WOMAC vs, 40% in CG), decrease pain (54% IG decrease four points vs, 39% in CG), reduce weight (54% lost 5% weight), and increase physical activity (24% decrease one category in IPAQ in a clinically relevant way (*P* <0.05). The adherence in the intervention group was 96% and in the control group 84%.

**Conclusion:** Preliminary results show that the coaching health intervention is effective for quality of life, pain, overweight, physical activity for people with KO compared to usual practice in a primary healthcare context in Barcelona.

# Abstract: The acceptability and feasibility of placing Sage advocates for vulnerable adults in the general practice setting

**Authors:** Anna Neary, Ealga Beary, Elizabeth McCarron, Leanne Hanrahan, Olivia Murphy, James Larkin, Pádraig Ruane, Bibiana Savin, Brendan O’Shea, Claire Collins, Aisling Ní Shuilleabhain

**Affiliations:** HSE/TCD GP Training Scheme, Dublin, Ireland;

## Abstract

**Background:** Research has shown reduced patient access to information regarding community-based services particularly within low-income households. Sage is an Irish advocacy service for older and vulnerable adults. Their work includes assisting people in maintaining control of their independence and supporting family members. General practice setting is well placed to identify suitable patients for referral to such a service.

**Research question:** To ascertain the feasibility and patient acceptability of the integration of an advocacy service into the general practice setting.

**Methods:** Five Irish general practices in the greater Dublin area have been introduced to a Sage representative and familiarized with the referral criteria and process. Patients are being selected and referred to the service by doctors or nurses in the practices. The quantitative analysis to be conducted in autumn 2017 will focus on the number of referrals and outcomes (e.g., attrition, number of meetings or referral elsewhere). Qualitative analysis will be based on structured interviews between practice teams and Sage representatives and three consenting patients per practice. Ethics approval has been granted by the TCD/HSE GP training scheme Ethics Committee.

**Results:** It is expected that uptake will vary between practices, due to practice size, demographics and GPs’ preferences. We anticipate an average of 20 referrals per practice within the four-month period. We will anticipate a positive response from those involved but will investigate any difficulties or practical issues to be highlighted by the qualitative analysis.

**Conclusion:** This study will pilot the introduction of a referral pathway; assess the response and experience of patients. Current research is limited in this field and this research may highlight the potential for basing advocates at primary care level. This is a multicentre study gathering data from patients with varying demographics results, and may form the basis for further study or for expanding the programme nationwide.

# Abstract: Validation of Patient Enablement Instrument (PEI) in Lithuanian general practice setting

**Authors:** Aelita Skarbaliene, Teresa Pawlikovska, Eva Lena Strandberg, Arnoldas Jurgutis

**Affiliations:** Klaipeda University, Klaipeda, Lithuania

## Abstract

**Background:** To provide better patient care outcomes, community-based primary care and patient-centred consultations are promoted. Increasing patient involvement both in their own care, and in its evaluation, is an important feature of contemporary health service development. The ideas of patient empowerment and patient enablement are especially emphasized nowadays. That is why Patient Enablement Instrument (PEI) was designed to capture patients’ feelings of understanding, confidence, and coping following a consultation. It can be used to evaluate core ingredients in primary care consultations for moving beyond patient satisfaction evaluations.

**Research question:** Validation of Patient Enablement Instrument in the Lithuanian context.

**Methods:** A quantitative questionnaire survey was carried out. The subjects were patients (*n* = 2500) consulting at the family doctors (*n* = 50) distributed within municipalities in Lithuania.

**Results:** Mean PEI score is 4.36 (out of 12). In addition, the questionnaire showed high internal consistency (Cronbach’s alpha 0.850). The results reveal that patient enablement increases with the consultation length (*P* <0.05), knowing the doctor (*P* <0.01) and getting a prescription when it is expected (*P* <0.01). Patient enablement decreases if patient prefers a different doctor (*P* <0.01) and where the doctor was interrupted in consultation (*P* = 0.05).

**Conclusion:** Since PEI validation studies were carried out in a few other counties before, there is a possibility to compare some results and obtain insights. In particular, mean PEI result in Lithuania is better than the results in Poland and the UK. At the same time mean, consultation duration is 14.3 min and is longer than the consultation duration in the Polish and UK studies—which is a feature of medical consultations that is usually good for patients. In general, the results of the research show the validity of PEI. Still further research would be of value.

# Abstract: Effect of physician notification regarding non-adherence to colorectal cancer screening on patient participation in faecal cancer screening: A randomized clinical trial.

**Authors:** Cedric Rat, Aurelie Gaultier, Jean-Michel Nguyen

**Affiliations:** Faculty of medicine, Nantes, France

## Abstract

**Background:** Increasing participation in faecal screening tests is a major issue in various countries that have implemented colorectal cancer (CRC) screening programmes. Developing interventions that are both cost-effective and non-time consuming is also challenging.

**Research question:** To determine whether providing general practitioners (GPs) a list of patients who are non-adherent to CRC screening enhances patient participation in faecal immunochemical testing (FIT).

**Methods:** A three-group, cluster randomized study was conducted from 14 July 2015 to 14 July 2016 in France. All the GPs of two French geographic areas were randomly assigned to one of three groups: 496 received a list of non-adherent patients (patient list intervention group, 10 476 patients), 495 received a letter describing region-specific adherence rates (generic reminder group, 10 606 patients), and 455 did not receive any reminders consistent (usual care group, 10 147 patients).

Eligible participants from GPs’ panels were adults aged 50 to 74 at average risk of CRC not up to date with CRC screening.

The primary endpoint was patient participation in CRC cancer screening one-year post-intervention.

**Results:** Among 1482 randomized GPs, 1446 completed the study; of the 33 044 patients of these GPs (mean age, 59.7 years old; 17 949 (54.3%) women), follow-up at one year was available for 31 229 (94.5%). At one year, patient participation per GP in FIT screening was 24.8% (23.4–26.2%) in the patient list intervention group, 21.7% (20.5–22.8%) in the group whose GP’s received generic reminders, and 20.6% (19.3–21.8%) in the usual care group, with between-group differences of 3.1% (1.3–5.0%) for intervention versus generic reminders, 4.2% (2.3–6.2%) for intervention versus usual care, and 1.1% (–0.6–2.8%) for generic reminders versus usual care.

**Conclusion:** Providing French GP’s with a list of their non-adherent patients resulted in a small but significant increase in patient participation in FIT screening for CRC compared with usual care.

# Abstract: Promoting influenza vaccination in general practice waiting rooms by posters and pamphlets. A registry-based cluster randomized controlled trial

**Authors:** Christophe Berkhout, Amy Willefert-Bouche, Emmanuel Chazard, Suzanna Zgorska-Maynard Moussa, Favre Jonathan, Lieve Peremans, Gregoire Ficheur, Paul Van Royen

**Affiliations:** Department of General Medicine, Medical School, Lille University, Lille Cedex, France

## Abstract

**Background:** Most general practitioners use advertising in their waiting rooms to educate patients. The number of patients vaccinated yearly against seasonal influenza has been gradually decreasing.

**Research question:** Our objective was to assess an advertising campaign for seasonal influenza vaccination using posters and pamphlets in GPs’ waiting rooms.

**Methods:** Registry based 2/1 cluster randomized controlled trial, a cluster gathering the listed patients over the age of 16 of 75 randomized general practitioners (GPs). The trial was conducted during the 2014–2015 influenza vaccination campaign. In the intervention group, 25 GPs received and displayed in their waiting rooms pamphlets and one poster (added to the usual mandatory information). In the control group (50 GPs); waiting rooms were kept in their general state. The main outcome was the number of vaccination units delivered in pharmacies. Data were extracted from the SIAM-ERASME registry of the Health Insurance Fund of Lille-Douai (Northern France). The association between the intervention and the main outcome was assessed through a generalized estimating equation.

**Results:** Seventy-five GPs participated in the study from 15 October 2014 to 28 February 2015, enrolling 10 597 patients over the age of 65 or suffering from long-lasting diseases (3781 in the intervention group; 6816 in the control group). No difference was found regarding the number of influenza vaccination units delivered (relative risk (RR) = 1.01; 95% confidence interval (95%CI): 0.97–1.05; *P* = 0.561). A vaccination performed on the previous year increased revaccination probability (RR = 5.63; 95%CI: 5.21–6.10; *P* <0.001).

**Conclusion:** Effects of the monothematic campaign promoting vaccination against influenza using a poster and pamphlets displayed in GPs’ waiting rooms could not be demonstrated.

# Abstract: Influence of patient characteristics on GPs advice on stopping statins in oldest-old: survey study across GPs from 30 countries.

**Authors:** Milly Anna Altena-Van Der Ploeg, Sven Streit, Nettie Blankenstein, Erna Beers, Arthur Bohnen, Daiana Bonfim, Huib Burger, Robert Burman, Tuz Canan, Claire Collins, Fabio Gazelato De Mello Franco, Margot De Waal, Biljana Gerasimovska-Kitanovska, Sandra Gintere, Raquel Gomez Bravo, Kathryn Hoffmann, Claudia Iftode, Kasper Johansen, Tuomas Koskela, Donata Kurpas, Hubert Maisonneuve, Christan Mallen, Christoph Merlo, Yolanda Müller, Christiane Muth, Martin Sattler, Sanda K Peštić, Ferdinando Petrazzuoli, Nicolas Rodondi, Thomas Rosemann, Tjard Schermer, Marija Petek Šter, Zuzana Švadlenková, Athina Tatsioni, Hans Thulesius, Victoria Tkachenko, Péter Torzsa, Rosy Tsopra, Bert Vaes, Marjan Van Den Akker, Rita Viegas, Shlomo Vinker, Katharine Wallis, Andreas Zeller, Jacobijn Gussekloo, Rosalinde Poortvliet

**Affiliations:** Leiden University Medical Center, Voorhout, Netherlands

## Abstract

**Background:** Statins are widely used to prevent cardiovascular disease (CVD). Current guidelines give little advice on when to stop statin treatment. GPs must rely on other factors like patient characteristics or common practice when making treatment decisions.

**Research questions:** Do patient-related characteristics (frailty, statin-related side effects, history of CVD or limited life expectancy) influence GPs’ advice to stop statins in the oldest old (>80 years)? Does advice vary across countries?

**Methods:** GPs from 30 countries (27 in Europe, Brazil, Israel, and New Zealand) participated in a survey based on case vignettes. We compared treatment advice in cases of >80 years varying in frailty, statin-related side effects, history of CVD and life expectancy <1 year. We compared the percentage of cases per GP that would be advised to stop. We used mixed-effect models to calculate odd ratio’s (ORsadj) adjusted for GP characteristics (such as gender) as fixed effects; country and GPs were adjusted as random effects.

**Results:** *n* = 2250. When no information about life expectancy was given, GPs advised stopping statin treatment in 46% of the cases (range: 23–58% across countries). When ‘life expectancy <1 year’ was added to case descriptions, GPs advised stopping statins in an additional 82% of the cases (range: 30–98% across countries). Frailty, side effects and life expectancy were associated with increased advice to stop (ORadj: 4.1; 95%CI: 3.8–4.4; ORadj: 1.62; 95%CI: 1.5–1.7 ORadj: 50.7; 95%CI: 45.5–56.4, respectively), while history of CVD was strongly related with decreased advice to stop (ORadj: 0.07; 95%CI: 0.07–0.08).

**Conclusion:** GPs were more likely to advise stopping statin treatment in >80 year-old patients when patients were frail, suffering statin side effects or had limited life expectancy; and more likely to continue statin treatment as secondary prevention for CVD. There were considerable treatment variations across countries.

# Abstract: Discrepancies in prostate-specific antigen-based screening for prostate cancer: a two-year follow-up cohort study of French men over the age of 40 years

**Authors:** Heloise Schmeltz, Cedric Rat, Sylvain Rocher, Aurelie Gaultier, Jean-Michel Nguyen

**Affiliations:** Faculty of Medicine, Nantes, France

## Abstract

**Background:** Prostate-specific antigen (PSA)-based prostate cancer screenings are controversial, resulting in discrepancies in physician practices.

**Research question:** The study objective was to identify factors related to the occurrence of patient PSA testing.

**Methods:** We performed a cohort study based on the French national healthcare system database (Loire-Atlantique geographic area). We reported the follow-up of a cohort of men aged over 40 years from 1 April 2014 to 31 March 2016. We first classified these patients according to their PSA testing status after two years of follow-up. We then analysed patient-related variables (i.e., age, low income, and variables illustrative of frailty, and comorbidities), using a multivariate mixed-effects logistic regression, and physician-related variables (i.e., gender, age, and practice characteristics) using logistic regression.

**Results:** Of 158 028 men (followed by 968 general practitioners), 32.3% (95%CI: 32.08–32.54%) men underwent PSA testing during the study period. PSA testing occurred less frequently in patients aged under 50 years (OR = 0.06; 95%CI: 0.06–0.07) or over 85 years (OR = 0.27; 95%CI: 0.25–0.29); patients with a low income (OR = 0.50; 95%CI: 0.45–0.55); frail patients characterized by the following conditions: the presence of a chronic disease (OR = 0.89; 95%CI: 0.86–0.92), repeated ambulance transportation (OR = 0.37; 95%CI: 0.31–0.44), or polymedication (OR = 0.90; 95%CI: 0.86–0.96); and patients with various specific comorbidities, such as cancer (OR = 0.80; 95%CI: 0.72–0.98), dementia (OR = 0.71; 95%CI: 0.59–0.85), or psychotic disorders (OR = 0.81; 95%CI: 0.74–0.88). There was a positive correlation between the patients’ probability of being screened and the number of men aged over 40 years in the physicians’ patient panel (*P* <0.001).

**Conclusion:** PSA-based screening remains frequent. It depends on the patients’ condition, frailty, and comorbidities.

# Abstract: General practitioner (GP)-centred care is effective in delaying the development of diabetes-related complications in Germany

**Authors:** Martin Beyer, Lorenz Uhlmann, Corina Guethlin, Marc S Hammer, Ferdinand M Gerlach, Karimova Kateryna

**Affiliations:** Univ. Frankfurt/M, Institute for General Practice, Frankfurt, Germany

## Abstract

**Background:** Through selective contracting in statutory healthcare, general practitioner (GP)-centred healthcare (HZV) is strengthened in Germany. We investigated the situation in the federal state of Baden-Württemberg. GP-centred care focuses on chronic diseases and includes elements of capitation fees, disease management, quality circles (peer review) and evidence-based care.

**Research question:** To compare the development of diabetes complications, measured in terms of clinical endpoints, of patients enrolled in GP-centred healthcare (HZV) and patients in usual GP care (non-HZV) over the course of four years (2011–14).

**Methods:** The primary endpoints in this time-to-event analysis were dialysis, blindness, amputation, stroke, myocardial infarction, cardiovascular disease, hypoglycaemia and mortality. For multivariable analysis, we used Cox proportional hazard regression models. Retrospective closed cohort study based on claims data.

**Results:** We included 217 964 patients in our study. In GP-centred healthcare 119 355 patients and usual GP care 98 609 were enrolled. Compared to routine care, the HZV group had a 15.6% lower risk of requiring dialysis during the four years of observation. We obtained similar results for all other endpoints except mortality.

**Conclusion:** The study indicates that GP-centred healthcare reduces the risk of diabetes complications. This may be because GP-centred care is associated with improved coordination.
